# Fabrication of UV Photodetector on TiO_2_/Diamond Film

**DOI:** 10.1038/srep14420

**Published:** 2015-09-24

**Authors:** Zhangcheng Liu, Fengnan Li, Shuoye Li, Chao Hu, Wei Wang, Fei Wang, Fang Lin, Hongxing Wang

**Affiliations:** 1 Key Laboratory for Physical Electronics and Devices of the Ministry of Education, Xi’an Jiaotong University, Xi’an, 710049, PR China

## Abstract

The properties of ultraviolet (UV) photodetector fabricated on TiO_2_/diamond film were investigated. Single crystal diamond layer was grown on high-pressure-high-temperature Ib-type diamond substrate by microwave plasma chemical vapor deposition method, upon which TiO_2_ film was prepared directly using radio frequency magnetron sputtering technique in Ar and O_2_ mixing atmosphere. Tungsten was used as electrode material to fabricate metal-semiconductor-metal UV photodetector. The dark current is measured to be 1.12 pA at 30 V. The photo response of the device displays an obvious selectivity between UV and visible light, and the UV-to-visible rejection ratio can reach 2 orders of magnitude. Compared with that directly on diamond film, photodetector on TiO_2_/diamond film shows higher responsivity.

UV photodetector is becoming increasingly important for its special applications in industry, instrument and our daily life such as flame detection, environment security, information technology, medical treatment and inter-satellite communication^1^. Since a high-performance photodetector should satisfy the 5S requirements known as high sensitivity, high signal-to-noise ratio, high spectral selectivity, high speed and high stability^2^, traditional UV-enhanced Si photodetector has some limitations in UV detection, for its bandgap energy is only 1.1 eV, which brings high cost filters and high temperature sensitivity^3^. Thus researchers began to develop photodetectors on wide bandgap semiconductors such as GaN^4^, ZnO^5^, SiC^6^ and Ga_2_O_3_^7^. Diamond also becomes an extraordinary candidate for ultraviolet photodetectors thanks to its wide band-gap, high carrier mobility, radiation hardness and thermal stability^8^. Earlier researches on diamond photodetector relied on natural diamonds, high-pressure high-temperature (HPHT) diamonds and polycrystalline diamonds. Recently, with the development of chemical vapor deposition (CVD) technique, high quality single crystal (SC) diamonds have been successfully grown onto low-cost diamond substrates^9^. And then, photodetectors with different structures on SC diamonds have been experiencing enthusiastic study, which shows highly desirable to satisfy the 5S requirements^10^,^11^. To realize a low dark current, as-grown diamond sample is oxidized to change the hydrogen termination surface into oxygen termination surface^2^. However, for undoped SC diamond epitaxial layer, the responsivity and UV-to-visible rejection ratio is relatively low when the surface is oxidized^2^.

TiO_2_ is another wide bandgap semiconductor, which is actively developed as detectors to be applied in the field of gas sensor, photocatalysis and solar cells^12^. Recently, TiO_2_ based photodetector has been fabricated by magnetron sputtering method, indicating very low dark current and high responsivity^13^,^14^. Thus, it is easy to think that combining TiO_2_ with diamond may provide a way to enhance the responsivity. Moreover, for a photodetector, when the photon energy is larger than the bandgap, the responsivity tends to decrease^15^. Considering that the bandgap of diamond is larger than that of TiO_2_, the combination of diamond and TiO_2_ may widen the spectral detecting range. This work is an attempt to deposit TiO_2_ directly on unintentionally doped SC diamond epitaxial layer to develop UV-photodetectors, whose optoelectronic characteristics have been investigated.

## Results and Discussion

**Figure 1:**
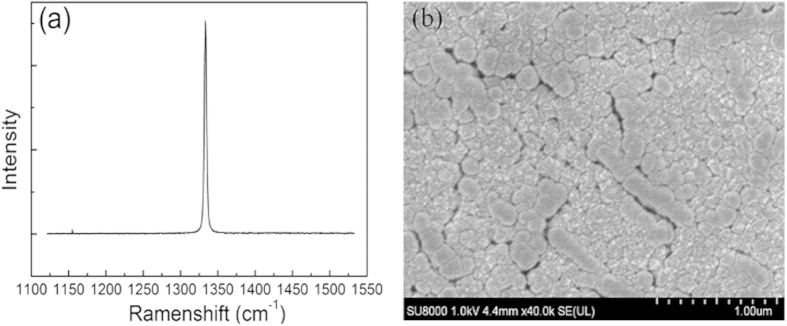
Characterization of diamond epitaxial layer and TiO_2_ film. (**a**) Raman spectrum of diamond epitaxial layer. (**b**) SEM image of TiO_2_ film.

Figure 1(a) shows the Ramen spectrum of the homoepitaxial diamond layer. There is only a sharp peak at 1332 cm^−1^ with a full width at half maximum of 3.9 cm^−1^, indicating a high-quality diamond layer^16^. Figure 1(b) exhibits the SEM image of TiO_2_ film sputtered on diamond layer and some interspaces exist between crystal grains. The TiO_2_ film may be polycrystalline. It was reported that oxygen partial pressure is important for depositing TiO_2_ film, because it may influence plasma potential, discharge voltage, and deposit rate^17^. In our deposition process, the partial pressure ratio of Ar to O_2_ was set as 2:1.

**Figure 2:**
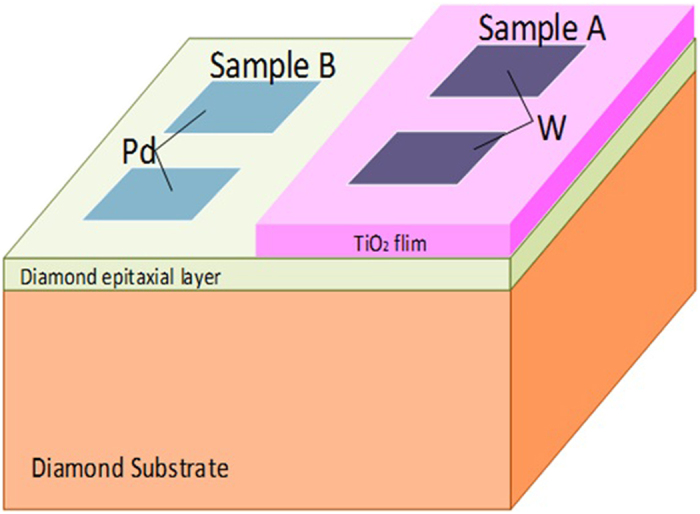
The schematic of device structures of Sample A and Sample B. The electrode width is 1 mm, and the interspace between two electrodes is 0.2 mm.

**Figure 3:**
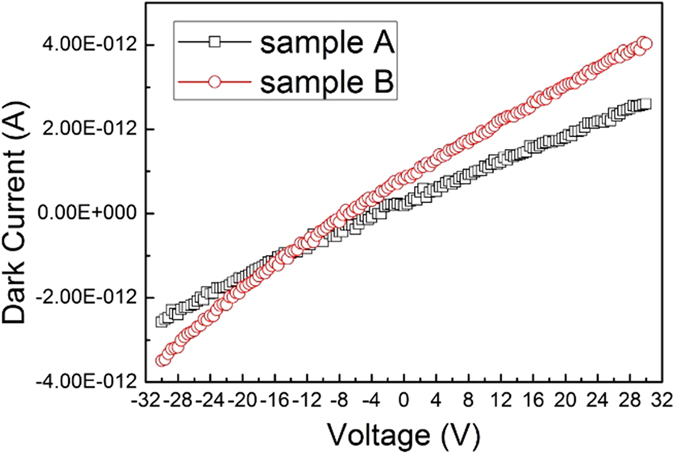
I–V Characteristics of Sample A and Sample B.

Figure 2 shows the schematic of photodetectors. The photodetector based on TiO_2_/diamond film is denoted as Sample A, and the other fabricated on diamond film is named as Sample B which is used for comparison. Since the electron affinity of TiO_2_ is about 4.3 eV, tungsten (W) with a work function of 4.55 eV was selected as electrode material in sample A to form ohmic contact. As for sample B, Pd was used to form ohmic contact. Dark currents of sample A and sample B were investigated and shown in Fig. 3. Both I–V curves are almost linear, showing that W/TiO_2_ and Pd/diamond contacts are both ohmic contacts. The dark current of sample A was measured to be 0.5 pA at bias voltage of 4 V. When the bias voltage increased to 30 V, the dark current increased slowly to 1.12 pA. For sample B, the dark currents are 1.28 pA and 1.9 pA at 4 V and 30 V, respectively. Both detectors show extremely low dark current, which plays an important role in lowering the signal to noise ratio^18^. Moreover, the dark currents are comparable, indicating that TiO_2_ film on diamond may not introduce additional leakage current path.

**Figure 4:**
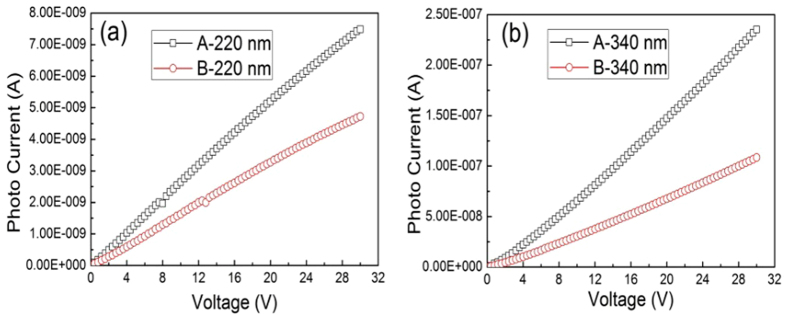
Photocurrents of sample A and Sample B under different illumination wavelengths. (**a**) The illumination wavelength is 220 nm, with a power density of 180 nW/mm^2^. (**a**) The illumination wavelength is 340 nm, with a power density of 23 μW/mm^2^.

The photocurrents of sample A and sample B were investigated by using UV-light with the parameter of 180 nW/mm^2^ at 220 nm and 23 μW/mm^2^ at 340 nm, as shown in Fig. 4. Compared to dark currents shown in Fig. 3, it indicates a significant increase of photocurrent in Fig. 4. For both detectors, photocurrents increase with bias voltage increasing, and no saturation phenomenon appears even at 30 V. A possible reason is that the electric field is not strong enough to collect all the photo-generated carriers before their recombination. Under the illumination of both 340 nm and 220 nm, the photocurrents of sample A are bigger than that of sample B, indicating a higher photo response of TiO_2_/diamond detector.

**Figure 5:**
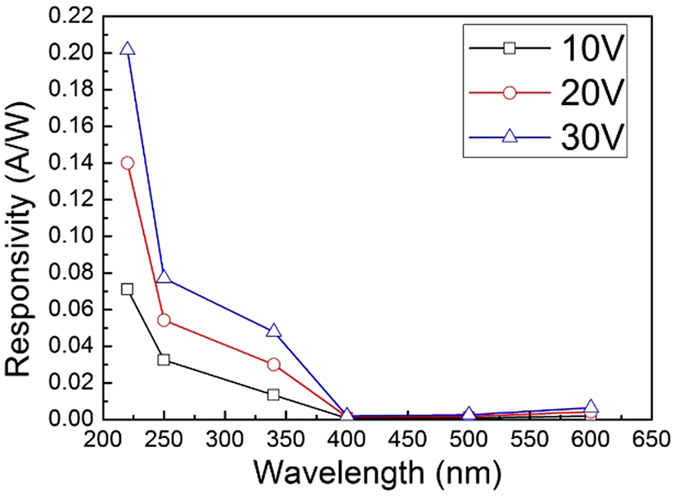
Spectral responsivity of Sample A at bias voltage of 10 V, 20 V and 30 V.

Figure 5 shows the responsivity of sample A varying with the wavelength changing under different bias voltages. The photo responsivity shows a decrease tendency with wavelength increasing. An obvious rejection ratio between UV and visible light can be observed. When the bias voltage is 10 V, under the illumination of 220 nm, 340 nm and 400 nm, the responsivity of sample A detector is calculated to be 0.071 A/W, 0.013 A/W and 0.00071 × 10^−4^A/W, respectively. Thus, the UV-to-visible rejection ratios for 340 nm versus 400 nm and 220 nm versus 400 nm are 18 and 100, respectively. When the bias voltage is increased to 30 V, the responsivity is evaluated to be 0.2 A/W, 0.048 A/W and 0.0019 A/W, leading to rejection ratios of 25 and 105, respectively. This indicates that when the bias voltage increases, the responsivity and rejection ratio also increase. The phenomenon is in agreement with the unsaturation of photocurrent. When higher electric field is applied, more photo-generated carriers can be collected, leading to responsivity enhancement. The detectivities of Sample A at 220 nm and 340 nm are 6.57 × 10^10^/W and 1.33 × 10^10^/W, respectively, indicating good UV detective ability.

**Figure 6:**
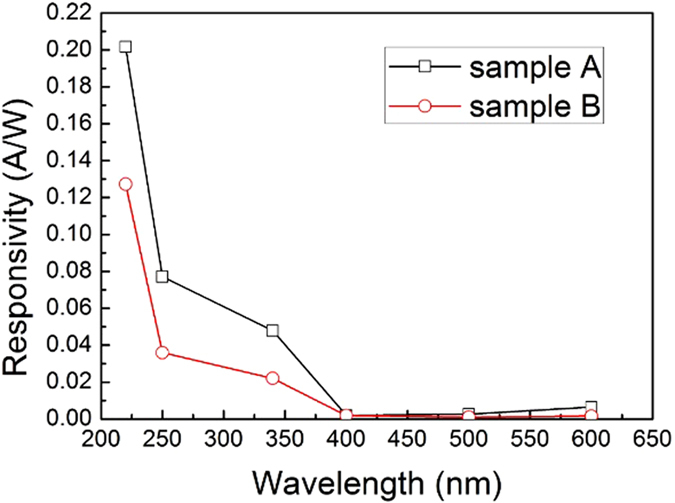
Spectral responsivity of Sample A and Sample B detectors at bias voltage of 30 V.

Figure 6 shows the spectral response of both detectors at 30 V, which displays the difference between TiO_2_/diamond detector (sample A) and diamond detector (sample B). For sample B, when the bias voltage is 30 V, under the illumination of 220 nm, 340 nm and 400 nm, the responsivity is calculated to be 0.13 A/W, 0.02 A/W and 0.0019 A/W, respectively. The UV-to-visible rejection ratio for 340 nm versus 400 nm is 11, while that for 220 nm versus 400 nm is 68. Compared to sample A, both of the responsivity and rejection ratio are lower. This means that the structure of TiO_2_/diamond can enhance the detector responsivity and rejection ratio.

**Figure 7:**
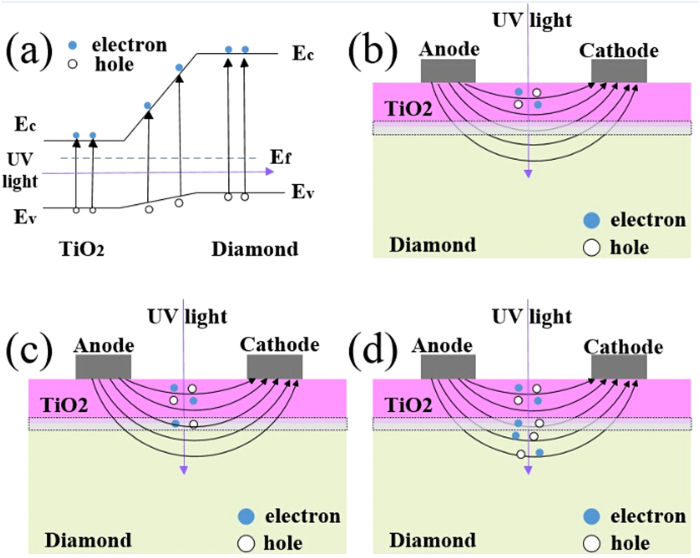
Schematic working principle of Sample A detector. (**a**) A rough schematic diagram of TiO_2_/diamond film. (**b**) Working principle of sample A under the illumination of 360 nm. The dotted area represents the TiO_2_/diamond interface, and the bend lines represent the electric field. (**c**) Working principle of sample A under the illumination of 225–360 nm. (**d**) Working principle of sample A under the illumination of less than 225 nm.

In order to investigate if sample A is depended on only TiO_2_ film, typical spectral responses of TiO_2_ detectors reported in references 13 and 19 are used for comparison. In reference 13, the highest responsivity is at a light wavelength of 250 nm, and the responsivity decreases when the wavelength is above or less than 250 nm. In reference 19, the highest responsivity is at 340 nm, and the responsivity tends to decrease when light wavelength becomes shorter. However, the spectral response of sample A detector is different, in which responsivity increases with the wavelength ranging from 400 nm to 220 nm. This phenomenon could be attributed to the TiO_2_/diamond joint film structure. When TiO_2_ is deposited on diamond, a gradient energy band would form in the interface, as shown in Fig. 7(a). When the incident UV light wavelength is shorter than cut-off wavelength, electrons can jump from the valence band to the conduction band. When the light wavelength is 360 nm, corresponding to the cut-off wavelength of TiO_2_, electron-hole pairs are generated in TiO_2_ film, as shown in Fig. 7(b). Then the carriers shift along the electrode field, and are collected by the electrodes. When the light wavelength is between 225 nm and 360 nm, the electron-hole pairs are generated both in interface and TiO_2_, as shown in Fig. 7(c), leading to an increase of photocurrent. When the light wavelength is shorter than 225 nm, which corresponds to the cut-off wavelength of diamond, electron-hole pairs are generated in TiO_2_, interface, and diamond, as shown in Fig. 7(d). Thus, more carriers contribute to photocurrent, resulting in a higher photo responsivity. For traditional TiO_2_ photodetector, it is fabricated only on TiO_2_ film is used as the works. Even though the light wavelength becomes shorter than cut-off wavelength, the amount of carriers remain the same, making no effect on improving the responsivity. In contrast, the responsivity may decrease, as shown in references 13 and 19.

**Figure 8:**
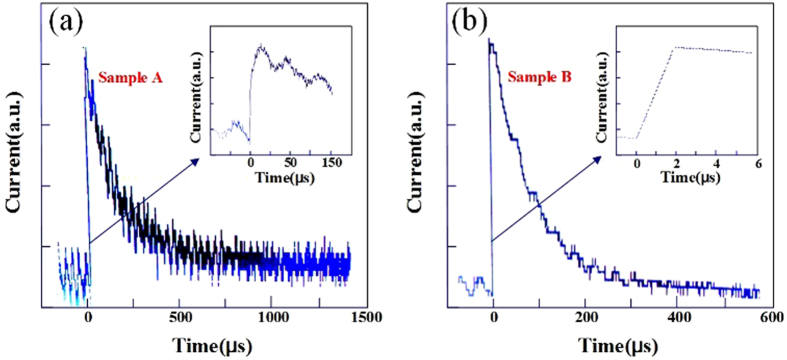
Temporal response behavior of Sample A and Sample B. (**a**) Temporal response of Sample A. (**b**) Temporal response of Sample B. Light pulse is induced by a 248 nm excimer laser, with a duration time of 50 ns and a frequency of 20 Hz. The insets show the rising speed.

Figure 8 shows the transient response of Sample A and Sample B under the illumination pulse of 248 nm light. The light pulse is generated by a KrF excimer laser, with a frequency of 20 Hz and a duration time of 50 ns. The insert show the increasing time of the photodetector. For Sample A, the increasing time is about 20 μs, and the decreasing time is about 1000 μs, as shown in Fig. 8(a). For Sample B, the increasing time is about 2 μs, and the decreasing time is about 400 μs, as shown in Fig. 8(b). For both photodetectors, the decreasing time is much longer than the increasing time, which is attributed to the delay in the decay of the photo-generated carrier density^16^. Compared to Sample B, the response time of Sample A is longer. The mechanisms could be considered as: one is that the oxygen molecules adsorption and desorption process on the TiO_2_ surface leads to the a slow response speed^20^; another is that the defects in the TiO_2_ film hinder the collection of some photo-generated carriers, leading to a longer response time^21^.

## Conclusions

In summary, the properties of ultraviolet photodetector fabricated on TiO_2_/diamond film have been investigated. TiO_2_ film has been directly deposited on single crystal diamond epitaxial layer by radio frequency magnetron technique. W electrodes were patterned on TiO_2_/diamond film to fabricate UV photodetector. This device exhibits 1.12 pA dark current at 30 V, and shows 2 orders of magnitude UV-to-visible rejection ratio. Compared with that of TiO_2_ photodetector, this device indicates increasing responsivity in a wide light wavelength range, which could be attributed to the gradient energy band structure in the interface of TiO_2_/diamond film. Also, the device shows higher responsivity than that on diamond. Transient response shows that the increasing time of the device is 20 μs and the decreasing time is 1000 μs.

## Methods

About 2 μm undoped SC diamond epitaxial layer was grown on 3 × 3 × 0.3 mm^3^ Ib-type HPHT diamond substrate by microwave plasma chemical vapor deposition method. The total flow rate of H_2_ and CH_4_, the ratio of CH_4_/(H_2_+CH_4_), the process pressure, the growth temperature and the microwave power were 500 sccm, 0.8%, 80 Torr, 850 °C and 800 W, respectively. Raman spectrum was used to characterize the quality of the epitaxial layer. After growth, the sample was boiled in acid mixture (H_2_SO_4_:HNO_3_ = 1:1 by volume) at 300 °C for 2 h to change the hydrogen terminated surface to oxygenated surface.

Half of this epitaxial layer was used to deposit TiO_2_ by radio frequency magnetron sputtering technique. The source material was 3 inches sintered TiO_2_ ceramic target with the purity of 99.99%. The background pressure was as low as 3 × 10^−4^ Pa. Ar and O_2_ were used as sputtering gas, whose flow rate were 40 sccm and 20 sccm, respectively. Before deposition, the target was cleaned by Ar ion for five minutes. During sputtering process, the power was 150 W, and the working pressure was 1.2 Pa. The thickness of TiO_2_ was measured to be 450 nm. Another half of diamond layer remained as oxygenated surface state.

Two W electrodes with a thickness of 100 nm were patterned on TiO_2_/diamond film using radio frequency magnetron sputtering method to fabricate the novel photodetector. The electrode width was 1000 μm and the space between electrodes was 200 μm. Thus the total active area was 0.2 mm^2^. Two Pd electrodes with a thickness of 50 nm were patterned on diamond film using thermal evaporation method to fabricate traditional diamond photodetector. These two electrodes had the same parameters as W electrodes. The I–V characteristics of the as-fabricated photodetectors were investigated by Agilent B1505A power device analyzer. The optoelectronic properties were evaluated with a Keithley 6487 picoammeter/voltage source, a 500 W Xe lamp source and a monochromator. The light power at the sample surface was measured by a commercial UV-enhanced Si detector. The time response is measured by a KrF excimer laser and an oscilloscope.

## Additional Information

**How to cite this article**: Liu, Z. *et al*. Fabrication of UV Photodetector on TiO_2_/Diamond Film. *Sci. Rep*. **5**, 14420; doi: 10.1038/srep14420 (2015).
